# The Transcription Factor HaHB11 Boosts Grain Set and Yield in Rice Plants, Allowing Them to Approach Their Ideal Phenotype

**DOI:** 10.3390/biom13050826

**Published:** 2023-05-12

**Authors:** Jesica Raineri, Luciano Nicolás Caraballo, Maximiliano Gómez, Raquel Lía Chan

**Affiliations:** Instituto de Agrobiotecnología del Litoral, CONICET, Facultad de Bioquímica y Ciencias Biológicas, Universidad Nacional del Litoral, Colectora Ruta Nacional 168 km 0, Santa Fe 3000, Argentina; jesica.raineri@santafe-conicet.gov.ar (J.R.);

**Keywords:** rice, GMO, HaHB11, HD-Zip I, idiotype, seed production

## Abstract

The ideal rice phenotype is that of plants exhibiting fewer panicles with high biomass, large grain number, flag leaf area with small insertion angles, and an erected morphology improving light interception. The sunflower transcription factor HaHB11, homeodomain–leucine zipper I, confers increased seed yield and abiotic stress tolerance to Arabidopsis and maize. Here, we report the obtaining and characterization of rice plants expressing *HaHB11* driven by its promoter or the 35S constitutive one. Transgenic *p35S:HaHB11* plants closely resembled the ideal high-yield phenotype, whereas those carrying the *pHaHB11:HaHB11* construct were hard to distinguish from the wild type. The former had an erected architecture, enhanced vegetative leaf biomass, rolled flag leaves with a larger surface, sharper insertion angles insensitive to brassinosteroids, and higher harvest index and seed biomass than the wild type. The combination of the distinct features exhibited by *p35S:HaHB11* plants, including the increased number of set grains per panicle, supports the high-yield phenotype. We wondered where *HaHB11* has to be expressed to achieve the high-yield phenotype and evaluated *HaHB11* expression levels in all tissues. The results indicate that its expression is particularly necessary in the flag leaf and panicle to produce the ideal phenotype.

## 1. Introduction

Rice (*Oryza sativa* L.) is one of the four main crops feeding the world’s population. It is in growing demand, challenging the scientific community to find strategies to enhance yields, stress tolerance, and nutritional quality. To improve these characteristics, it is necessary to have deep knowledge of plant physiology and molecular biology. In addition to the crucial role of rice in global food security, it also serves as a model for monocotyledonous species [1]. Knowledge of this species was significantly advanced in recent decades, revealing several architectural parameters defining seed production.

Regarding seed production, the traits associated with increased yield are large panicle size, moderate tillering capacity, heavy and drooping panicles at maturity, erected leaves until maturity, flag leaf length above panicle height, small flag leaf–uppermost internode angle, and a harvest index equal to or higher than 0.55. The combination of these characteristics in a single plant constitutes the ideal high-yield phenotype [2].

Some architectural parameters significantly impact rice yield. The height of the uppermost internode is one of them. Short uppermost internodes cannot push the panicle out of the flag leaf, resulting in panicle enclosure [3]. Panicle enclosure blocks normal development and pollination, reducing seed production in hybrid rice. This characteristic was associated with IAA and GA contents, affecting cell number and elongation [3]. Tiller diameter and height are also related to grain yield, constituting the main characteristics selected during the Green Revolution, because shorter plants spend less energy producing tillers, which can help to improve lodging resistance, and are less susceptible to the overturning produced by strong winds. The erectness of leaves represents an important agronomic trait. During ripening, the leaf inclination angle diminishes. Notably, the cultivar Takanari (high-yield indica) exhibits more erected leaves than Koshihikari (high-quality japonica). Cells on the flag leaf adaxial side significantly increased after leaf expansion in the japonica cultivar, whereas no significant differences were observed in indica, indicating that cell elongation causes the differences in the leaf inclination angle [4]. Kitaake is a model rice variety easy to propagate, short in stature, having a nine-week life cycle. This variety can be routinely transformed and edited, and multiple insertional mutants in this background are available, as well as a public database offering protocols and genetic tools (https://kitbase.ucdavis.edu/home; accessed on 8 April 2023 [5]). 

Considering leaf abaxial curling, the insertional mutant BY240 exhibits a differential phenotype. This mutant, obtained with T-DNA insertion interrupting the ACL1 (Abaxially Curled Leaf 1) gene promoter, leads to the overexpression of BY240, resulting in leaf curling. ACL1 has a homolog, ACL2, and both participate in the leaf morphology architecture. A microscopical analysis indicated more bulliform cells and the expansion of epidermal cells. A discoordination of the abaxial and adaxial leaf sides resulted in abaxially curled leaves [6]. 

Among the strategies to improve rice and other crops’ yield and quality, one frequently used is the alteration of certain regulatory protein levels. There are many examples of the modulation of transcription factor (TF) expression by means of transformation or editing that became biotechnological tools. TFs can repress or induce whole signaling pathways. In plants, TFs were classified into several families and subfamilies, mainly according to their DNA binding domain and other characteristics, such as the genetic structure and additional functional motifs [7,8]. Among them, the homeodomain–leucine zipper family is composed of four groups, I to IV; their members exhibit the unique association of a homeodomain (HD), which recognizes and binds specific DNA sequences, and a leucine zipper (LZ), able to homo- or heterodimerize. The dimerization of these proteins is a prerequisite to DNA binding [9]. Aside from the HD-Zip domain, these TFs exhibit uncharacterized conserved motifs in their carboxyl and amino termini [10]. Among these motifs, only the transactivation AHA motif was assigned a function so far [11]. Several of these motifs interact with other components of the transcriptional regulatory machine [11]. For example, Arabidopsis member AtHB23, involved in root development and salinity response, interacts with MYB TF AtPHL1 and AtMYB68 [12,13].

In rice, thirty-one transcription factors belonging to HD-Zip families I, II, and III were identified after a genome-wide survey aiming at determining drought-responsive genes. Among them, thirty were expressed in mature plants and seedlings. Types I and II, as in Arabidopsis, were related to water deficit response [14]. Besides OsHOX4, studied in the same work, only a few were functionally characterized. HOX12 is involved in panicle exertion as a positive modulator of EUI1 (ELONGATED UPPERMOST INTERNODE1; [15]). OsHOX1 and OsHOX28, members of the HD-Zip II subfamily, severely repress the expression of HEAT STRESS TRANSCRIPTION FACTOR2D (HSFA2D), the main responsible for tiller angle establishment in rice and the local distribution of auxin [16]. A member of subfamily IV, TF1L, is expressed in rice, conferring drought tolerance, promoting lignin biosynthesis and stomatal closure [17]. Aside from those of rice, many HD-Zip TFs from varied species have been studied and reported as crucial players in developmental events and environmental responses [9,18]. 

HaHB11 is a divergent member of the HD-Zip I sunflower subfamily. Similar to its sister, HaHB4, this TF exhibits a very short carboxyl terminus. These HD-Zip I members only exist in the Asteraceae superfamily and are absent in the genomes of the model and crop species. When introduced in Arabidopsis, HaHB11 interacted with the motor protein Kinesin13B; such interaction was crucial to conferring a differential phenotype [19]. Besides Arabidopsis, this sunflower gene was ectopically expressed in alfalfa and maize plants, triggering flooding tolerance and enhanced seed yield [20,21]. In particular, the expression of *HaHB11* in maize significantly augmented the grain set in lines and hybrids under normal conditions or under stress caused by waterlogging or defoliation [22,23]. Notably, among the differential traits produced by this TF and also by HaHB4, the enlargement of stem width was associated with increased yield in Arabidopsis and sunflower plants [24]. 

Here, we show that in five field trials, *HaHB11* conferred several differential characteristics to rice plants that allowed them to approach the ideal phenotype. Rice plants transformed with *35S:HaHB11* exhibited higher leaf biomass, more grains, enhanced flag leaf area with smaller insertion angles, and an erected morphology compared with their controls or transgenic plants transformed with the construct *pHaHB11:HaHB11*.

## 2. Materials and Methods

### 2.1. Plant Material, Growth Conditions, and Treatments

Greenhouse conditions: Rice plants from the genotype Kitaake were grown in 8 L pots under a long-day photoperiod (16/8 h light/dark cycles). For each genotype and condition, at least five biological replicates were included in the assay. Daily temperatures fluctuated between a minimum of 10 °C and a maximum of 40 °C, depending on the month of the assay. Although the temperature reached almost 40 °C on some days, all plant genotypes were grown together and exposed to the same conditions. Plants were well irrigated during their life cycle and fertilized once a week with Hakaphos^®^ Rojo and Basafer Plus^®^. The multiplication of seeds was performed in the greenhouse.

Field trial conditions: Five independent experiments were conducted every year between 2017 and 2021 at Institute of Agrobiotechnology (IAL) located in Santa Fe (31°38′17.1″ S, 60°40′01.8″ W) in pools (nylon-isolated, making flooding possible) of 2 m in width, 5 m in length, and 30 cm in depth. The evaluated germplasm included Kitaake controls and transgenic lines transformed with *35S:HaHB11* (independent events H11-1 and H11-2) and *proHaHB11:HaHB11* (independent events pH11-2 and pH11-3). In the first campaign, we used null segregants as controls; since the results were similar to those obtained with the wild-type genotype, we continued with the latter. Sowing took place in November of every year at the single-stand density of 223 plants m^−2^. Such density was decided after observing the short stature of the Kitaake variety and the planting uses for japonica [25]. The three genotypes were distributed in a completely randomized design with three replicates of each one. Parcels had three–four rows of 2 m in length. The distance between rows was 15 cm, and between individual plants, 3 cm. The plants were drip-irrigated daily until flooding, and urea was applied as fertilizer (50 kg/ha) 15 and 50 days after sowing (DAS). The trials were kept free of weeds, insects, and diseases by employing the necessary chemical and manual controls and were flooded when seedlings reached the V5 stage (five fully expanded leaves). The results presented in the figures are representative of the five repetitions. The sowing, waterlogging, anthesis, and harvest dates of each trial are listed in Supplementary Table S1. IRGA is a commercial genotype included as control in several trials. All assays were carried out after obtaining the corresponding authorization from CONABIA (National Committee of Biotechnology) and INASE (Seeds National Institute).

### 2.2. Genetic Constructs

*35S:HaHB11*: The complete cassette was restricted with HindIII and EcoRI from a clone (pBI 121) used to transform Arabidopsis plants [20] and cloned in the same sites of pCAMBIA1308. Plants transformed with this construct were named H11.

*ProHaHB11:HaHB11*: The entire segment was restricted from the pBI construct [20] with HindIII/EcoRI and subcloned in pCAMBIA 1308 in the same sites. This fragment contained a 1373 pb sequence upstream of the transcription start site of the sunflower *HaHB11* promoter plus its cDNA. Plants transformed with this construct were named pH11.

### 2.3. Rice Transformation

Agrobacterium-mediated rice callus transformation was essentially performed as described by Main et al. [26] with few modifications. The agrobacterium tumefaciens EHA105 strain was used and transformed with the binary plasmid pCAMBIA1380 containing a *35S:HaHB11* or *pHaHB11:HaHB11* expression cassette. Mature Kitaake rice seeds were surface-sterilized and germinated in callus induction medium containing 2,4-D (10 mg/mL). Embryogenic calli (white and friable) were selected and subcultured in fresh induction medium three days before infection. Calli were infected with Agrobacterium for two minutes and co-cultivated on wet filter paper for three days in the dark. Calli were then transferred to selection medium containing 50 mg/L hygromycin for transgenic callus selection, timentin (100 mg/mL), and cefotaxime (100 mg/mL) for Agrobacterium growth control and incubated in the dark at 30 °C. Actively growing calli in the selection medium were individualized and changed to regeneration medium. Plates were sealed with vent tape and placed in the light (16:8 h photoperiod, 80–100 μmol/m^2^/s, 28 °C) until shoots developed. Plantlets were moved to jars containing 45 mL of medium for further root elongation. The shoots exhibiting an adequate root system were placed in a growth chamber and subsequently transferred to the greenhouse. 

### 2.4. RNA Isolation and Expression Analyses with Real-Time RT-PCR

Total RNA for real-time RT-PCR was isolated from rice tissues using Trizol^®^ reagent (Invitrogen, Carlsbad, CA, USA), and real-time qPCR was performed using an Mx3000P Multiplex qPCR system (Stratagene, La Jolla, CA, USA) as previously described [20]. The primers used are listed in Supplementary Table S2. 

### 2.5. Crop Phenotyping

Crop phenotype was assessed during the five field trials during spring–summer 2017–2021. Samples were taken from at least six plants per plot and evaluated from day 30 after sowing until harvest. Tillers, vegetative leaves, flag leaves, and panicles were manually counted. Rolled and unrolled leaf length and width were measured with a ruler. Plant height was considered from the soil surface to the uppermost visible node (tiller length—UI), and tiller diameter was measured at the base of the plant. Vegetative (tillers, vegetative leaves, and flag leaves) and reproductive (panicles) biomass was obtained after oven-drying at 60 °C until the sample weight was constant (over approximately three days). The uppermost internode length was considered from the base of the panicle to the node directly below it.

The anthesis day was registered when 50% of the plants in the plot exhibited visible hanging anthers having mature pollen. 

Flag leaf and tiller angles were quantified using a protractor in situ. Alternatively, we took pictures of individual tillers and used the free ImageJ software.

Plants from all the genotypes, occupying 50 linear cm of each row, were harvested and evaluated 115 DAS, which was considered the end of the life cycle. Grain yield (panicle weight) was assessed in these samples, and the harvest index (HI) was calculated as the quotient between grain yield and total plant biomass. Width, height, and length were determined for individual grains. Branching was evaluated by dissecting the panicles, and grain number was assessed in each branch. 

The evolution of light interception was obtained fortnightly between 31 and 93 DAS. It was computed as in Equation (1) [27].

Light interception = 1 − (IPARb/IPARa)(1)

where IPARa and IPARb represent incident photosynthetically active radiation above the canopy and immediately below the lowermost green leaves, respectively. 

### 2.6. Green Index Evaluation 

The green index was determined using a specific chlorophyll meter device (Cavadevices^®^, Buenos, Aires, Argentina^;^ https://cavadevices.com/archivos/FOLLETOS/Clorofilio.pdf; accessed on 8 April 2023). Values were expressed as chlorophyll content index (CCI), calculated using the following formula: CCI = %T940 nm/%T660 nm, where % TXX nm means the transmittance through the leaf at the specified wavelength.

### 2.7. Histology

Histology of the cross-sections was carried out as previously described [22], and cross-sections were stained with safranine fast green. Cross-sections were evaluated using microscopy. Photographs were taken with a Nikon Coolpix L810 camera mounted on an Eclipse E200 microscope (Nikon, Tokyo, Japan).

Large vascular parenchyma sections, bulliform cell number, flag leaf area, large vascular bundle diameter of flag leaf and tiller, the transversal section area of the uppermost internode, and tiller thickness were assessed using the program ImageJ (1.50 v) [28]. 

### 2.8. Brassinosteroid Treatment 

The brassinosteroid effect on flag leaf angle was evaluated as described by Li et al. [29] with a few modifications. Detached vegetative leaves were incubated in darkness at 28° in deionized water or with 1 × 10^−7^ M brassinolide (BL) addition. A minimum of 12 leaves were used per point. Photographs and samples were taken two days after initiating treatment.

### 2.9. Sequence Analyses

The HaHB11 cDNA sequence was compared with the rice genome database using the Blast tool. Moreover, we used an optimized algorithm to compare two similar sequences [30]. 

### 2.10. Statistical Analyses

As indicated in the corresponding figure legends, ANOVA followed by Tukey’s (differences across means) tests were used for the comparison of all the parameters evaluated across field experiments. Error bars represent SEM (standard error of the mean). *p*-Values are indicated in the legends of the corresponding figures. PCA was used to evaluate the correlations among traits for the different genotypes. 

### 2.11. Accession Numbers

The accession numbers of the genes evaluated in this work are available in Supplementary Table S2.

## 3. Results

### 3.1. Rice Plants Constitutively Expressing HaHB11 Partition More Biomass to Leaves Than Controls and Exhibit Delayed Anthesis

**Figure 1:**
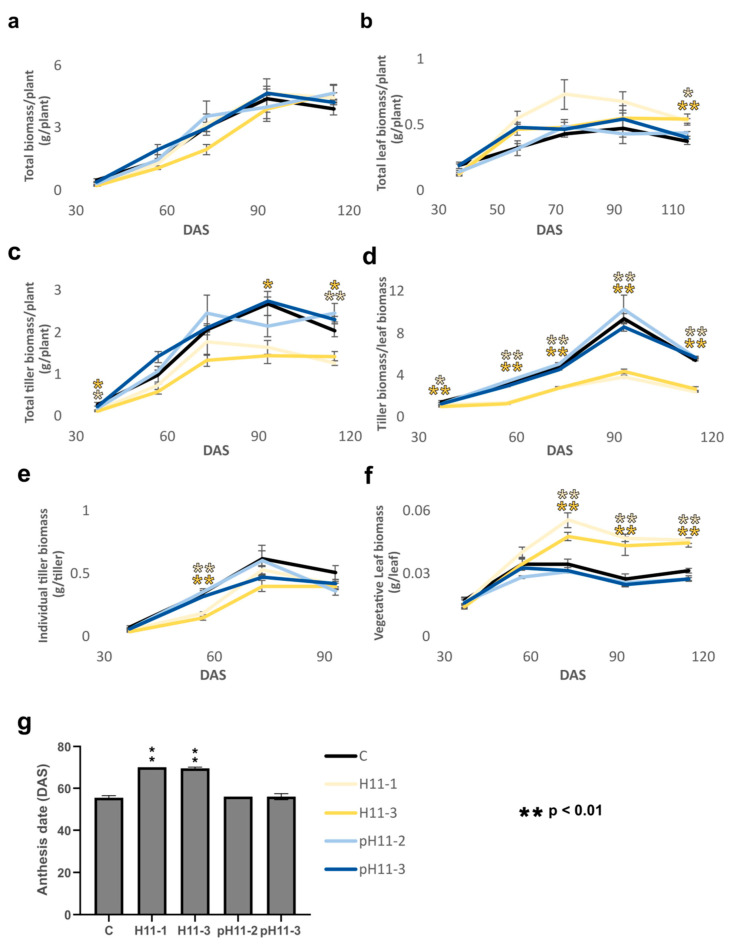
Rice plants constitutively expressing *HaHB11* partition more biomass to leaves than to tillers and exhibit delayed anthesis. (**a**) Total biomass per plant. (**b**) Total leaf biomass per plant. (**c**) Total tiller biomass per plant. (**d**) Total tiller biomass/total leaf biomass ratio. (**e**) Individual tiller biomass. (**f**) Individual vegetative leaf biomass expressed in g/leaf. (**g**) Anthesis day. Assays were repeated during four campaigns with N: 18–24/genotype. Wild-type Kitaake plants were used as controls (C) and are graphed in black color, whereas H11 and pH11 plants are represented in yellow and blue, respectively. The figure illustrates the data obtained in one of these campaigns, that of summer 2021–2022. The whole data of the five campaigns can be visualized in Table S3. Error bars represent SEM. Asterisks indicate significant differences (* *p* < 0.05, ** *p* < 0.01).

*HaHB11* expression in Arabidopsis and maize plants enhanced their biomass and seed yield. Because rice plants are evolutionarily distant from those species, we wondered whether the sunflower gene could confer beneficial traits to this species. HaHB11 is a divergent member of the sunflower HD-Zip I family of TFs and has no homologs outside the Asteraceae group [10]. Sequence comparison in public databases indicated that its closest rice member is OsHOX6. However, this TF strongly differs from HaHB11, particularly outside the HD-Zip domain in the N and C termini. Global identity is lower than 30%, whereas similarity is around 50% (Supplementary Figure S1). We generated transgenic rice plants with two constructs: one in which the *HaHB11* promoter drove the *HaHB11* cDNA expression (events pH11-2 and pH11-3) and the second carrying the constitutive 35S mosaic virus (events H11-1 and H11-3). Two independent lines from each construct and their controls were grown in large pools in open air and evaluated during the life cycle. In the early vegetative stage, the growing rate of H11 plants was slower than that of the other genotypes, accelerating after 70 DAS (Figure 1a). Tiller and leaf biomass was weighed from day 30 after sowing to harvest. Total biomass per plant was similar in all the genotypes (Figure 1a), whereas total leaf biomass per plant was slightly larger in H11 plants (Figure 1b), and total tiller biomass per plant significantly decreased in the same plants compared with controls and pH11, particularly at the end of the life cycle (Figure 1c). During almost the entire life cycle, H11 plants partitioned more biomass to leaves than tillers (Figure 1d). On day 93, individual leaf biomass was higher in H11 plants, resulting in enhanced total leaf biomass, although leaf biomass per plant tended to be lower (Supplementary Figure S2c). Individual tiller biomass was lower in H11 plants on day 53 but did not show significant differences later or at harvest (Figure 1e), while individual vegetative leaf biomass was significantly larger in H11 plants than in controls and pH11 (Figure 1f). Neither the number of leaves per plant nor the green index in vegetative and flag leaves exhibited significant differences among genotypes (Supplementary Figure S2a,b,d,e). Although the number of tillers, and in consequence that of flag leaves, was smaller in H11 plants, flag leaf biomass in these plants almost doubled that of controls and pH11 plants. This is because each one was larger and heavier than those of the other genotypes. (Supplementary Figure S2c). Tillering capacity on day 93 was around 750 for H11 plants, whereas controls and pH11 plants had 950 and 1300 panicles/m^2^, respectively. Together with these characteristics, H11 plants exhibited an anthesis delay of approximately 15 days compared with the wild-type genotype, whereas pH11 did not differ from controls, such as in the other evaluated characteristics (Figure 1g, and Supplementary Tables S1 and S3). However, the total life cycle did not significantly differ among genotypes, and harvest was carried out on the same date for all the plants (Supplementary Tables S1 and S3), indicating that the grain-filling stage was shorter in H11 plants than in controls. Altogether, these results suggest that widely expressed *HaHB11* triggered differential biomass partitioning to leaves.

### 3.2. In the Reproductive Stage, H11 Plants Exhibit Significantly Improved Traits Compared with Controls and pH11 Plants

**Figure 2:**
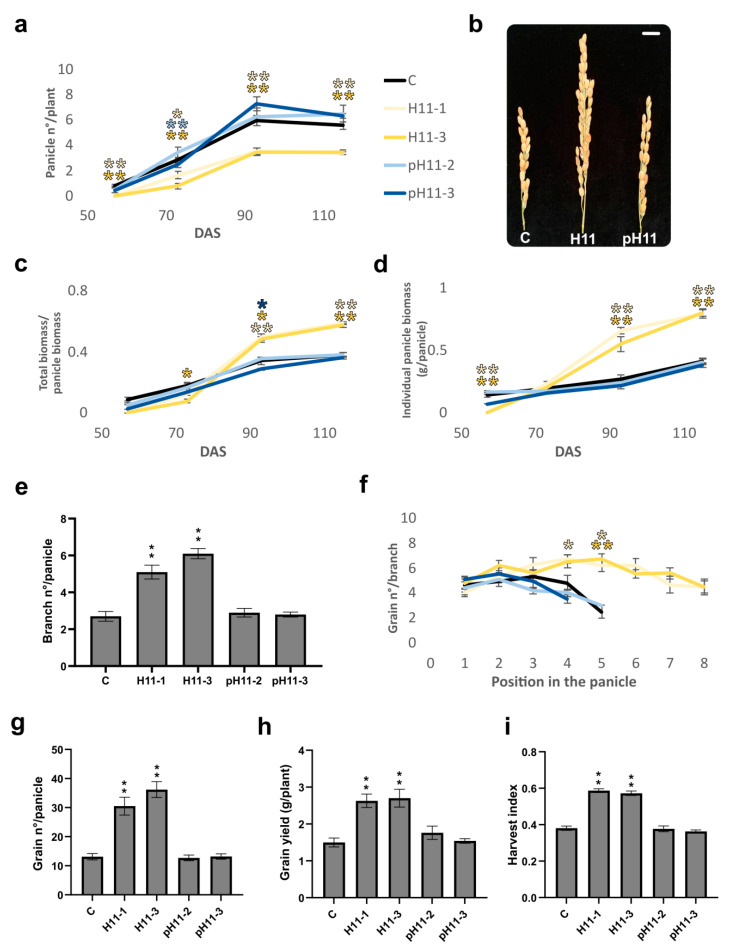
H11 plants have fewer panicles per plant, albeit heavier, and produce more grains than controls. (**a**) Number of total panicles per plant. (**b**) Illustrative picture of panicles at maturity; the scale bar represents 1 cm; C, H11, and pH11 plants. (**c**) Total biomass per panicle biomass ratio. (**d**) Individual panicle biomass. (**e**) Number of branches per panicle. (**f**) Number of grains per branch. (**g**) Number of grains per panicle. (**h**) Individual plant seed yield. (**i**) Harvest index. The trials were repeated at least three times with N: 18–24/genotype. The figure illustrates the data obtained in one of these campaigns, that of summer 2021–2022. The whole data of the five campaigns can be visualized in Table S3. Error bars represent SEM. Asterisks indicate significant differences (* *p* < 0.05, ** *p* < 0.01).

**Figure 3:**
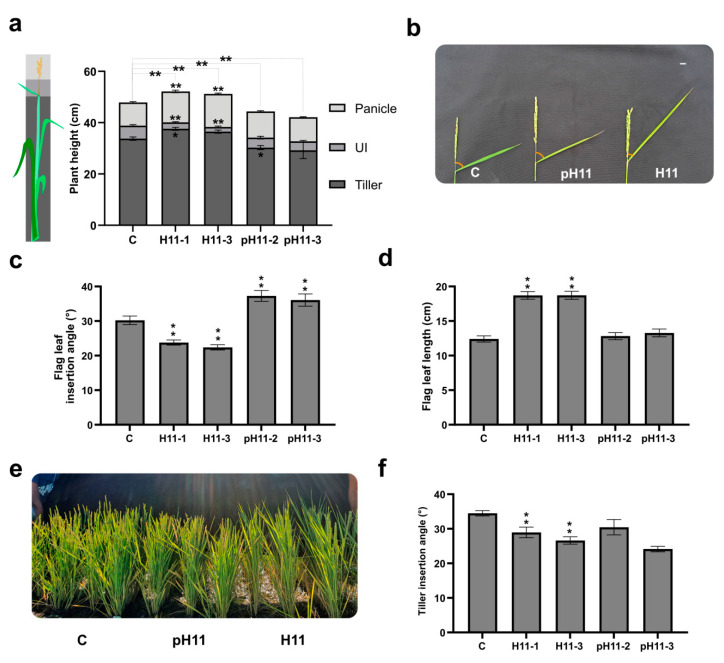
H11 plants exhibit a differential architecture impacting seed yield. (**a**) Plant height on day 93 after sowing, indicating the contribution of the tiller, the uppermost internode, and the panicle length. (**b**) Picture illustrating the differences between genotypes in uppermost internodes and flag leaf angles of 93-day-old plants; measured angles are shown in red. (**c**) Angle formed between the uppermost internode and the flag leaf seven days after flowering. (**d**) Flag leaf length in 97-day-old plants. (**e**) Illustrative picture of control, pH11, and H11 93-day-old plants. (**f**) Tiller angles assessed 30 days after sowing. Assays were repeated at least three times with N: 18–24/genotype. The figure illustrates the data obtained in one of these campaigns, that of summer 2021–2022. The whole data of the five campaigns can be visualized in Table S3. Error bars represent SEM. Asterisks indicate significant differences (* *p* < 0.05, ** *p* < 0.01).

We assessed traits directly associated with seed yield to evaluate the impact of the differential characteristics described above. Notably, the number of panicles per plant was lower in H11 plants than in the other genotypes (Figure 2a). However, such panicles were significantly longer and wider and almost doubled the weight of those in controls and pH11 plants (Figure 2b and Figure S3b, and Tables S3 and S4), except for one of the pH11 lines (pH11-2), which had longer panicles than controls, albeit shorter than H11 (Figure 3a). Individual panicle biomass and the ratio of panicle biomass/total biomass were higher in H11 plants than in controls (Figure 2c,d). The analysis of individual panicles indicated that they had more branches, and each one had more grains than controls and pH11 plants (Figure 2e,f and Figure S3b). Grain size and weight, individual width, height, and length, as well as 100-grain weight, did not differ among genotypes (Supplementary Figure S3c and Tables S3 and S4), whereas the number of panicles per tiller was lower in H11 plants (Supplementary Figure S3e). However, H11 plants stopped tillering earlier than the other genotypes and sent their resources to panicles. Notably, aerial biomass partitioning significantly differed in H11 plants (Supplementary Figure S3d), supporting their shorter stage of panicle filling, which compensated for the anthesis delay, leading to life cycle similarity with the other genotypes. Remarkably, one of the crucial traits determining seed yield, the harvest index, was significantly higher in H11 plants than in controls and pH11 lines (Figure 2h). It is important to note that the harvest index was calculated by only considering the aerial biomass. The combination of differential traits, particularly those shown by panicles (Figure 2g), resulted in an enhanced number of grains per panicle, positively impacting seed yield (Figure 2g).

### 3.3. The Uppermost Internode Length, Flag Leaf, and Tiller Angle in H11 Plants Contribute to Explaining Their Increased Yield

The ideal phenotype of rice plants is a set of several combined traits that together lead to better grain set, harvest index, and grain yield. 

We evaluated these characteristics in *HaHB11*-modified plants. Uppermost internodes were shorter in H11 than in control plants (Figure 3a,b and Figure S4a). Regarding flag leaf angle, it was sharper in H11 plants and more open in pH11 plants than in the wild type (Figure 3b,c). Notably, individual tiller length was longer below the uppermost internode in H11 plants and shorter in pH11 ones than in controls. H11 plants lost this difference when the total length of tillers was assessed, because the uppermost internode was shorter, compensating for the total length. On the other hand, pH11 lines retained the differential trait (Figure S3a). Flag leaf laminas were longer in the H11 genotype, while pH11 did not significantly differ from controls (Figure 3d). These characteristics were easy to visualize (Figure 3e and Figure S4c). Light interception did not differ among genotypes (Supplementary Figure S4b), but H11 plants exhibited a leaf-rolled structure, and the top three leaves were above the panicle height, impacting the exposed leaf area (Figure 3b and Figure S4c). Remarkably, tiller angles were sharper in the H11 genotype and pH11 plants (one out of two lines and a tendency in the second one). Despite their differential leaf architecture, light interception in H11 plants was similar to that in controls (Supplementary Figure S4b,d). We carried out a principal component analysis considering H11 and control plants (Supplementary Figure S4d), supporting the observations described above.

Given that *HaHB11* encodes a transcription factor and modulates the expression of endogenous genes related to the observed differential traits, we quantified, in the panicle and flag leaf, the transcript levels of well-characterized genes previously assigned a role in the rolled leaf phenotype and enhanced yields. *OsROC5* and *OsAGO1* were slightly induced in H11 and pH11 panicles and did not differ in flag leaves, whereas *OsHOX32* did not show modulation in these tissues, nor did *OsPFL* in panicles, and the latter was not detected in flag leaves (Supplementary Figure S5).

### 3.4. H11 Plants Exhibit Larger Vascular Bundles in Flag Leaf and Tiller Than Controls

**Figure 4:**
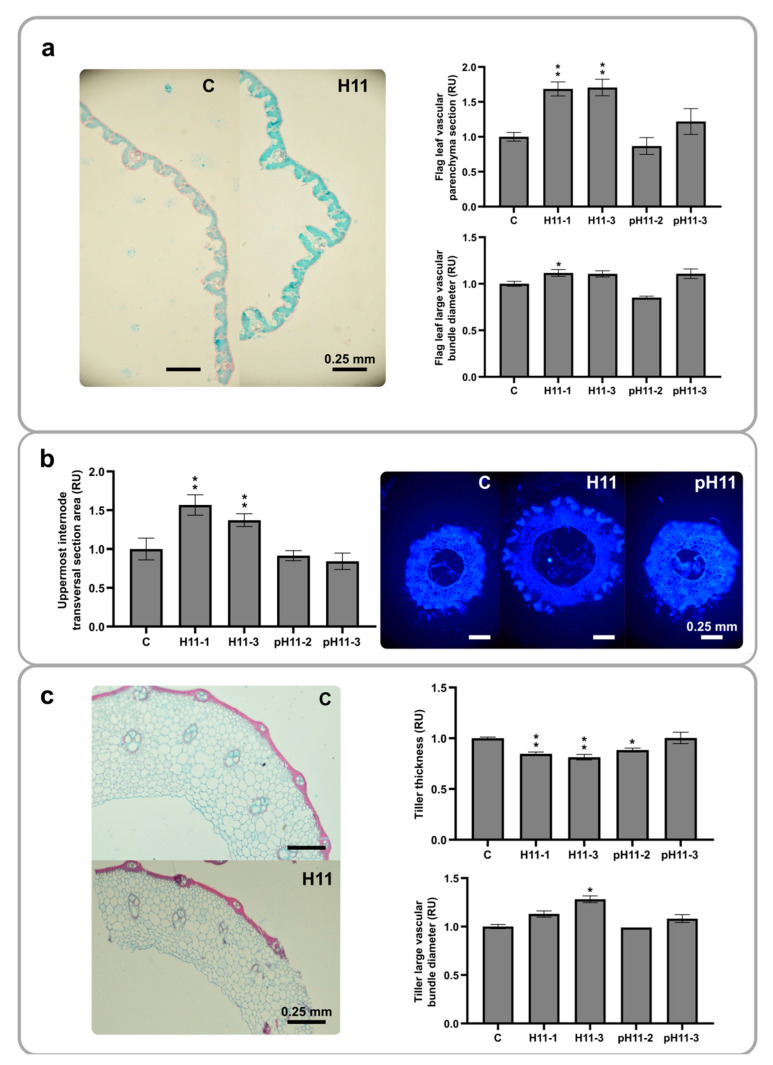
Vascular bundles in the flag leaf and the uppermost internode are larger in H11 plants than in controls. (**a**) Left panel: illustrative picture of flag leaf histological section from control and H11 93-day-old plants; right panel: quantification of flag leaf vascular parenchyma surface and flag leaf large vascular bundle diameter. (**b**) Left panel: transversal section area of the uppermost internode; right panel: illustrative pictures of uppermost internode sections of 93-day-old plants. (**c**) Illustrative pictures of tiller transversal sections of 93-day-old plants; right panel: quantification of tiller thickness and large vascular bundle diameter. All the values were expressed in relative units (RUs) after normalizing them to those obtained in control plants (C), which were arbitrarily assigned a value of 1 (one). The assays were repeated at least twice with N: 4–6/genotype. Error bars represent SEM. Asterisks indicate significant differences (* *p* < 0.05, ** *p* < 0.01).

Regarding the differential traits described so far, we were interested in whether the cellular organization at the microscopic levels of leaves and stems could explain the macroscopic characteristics. For this purpose, flag leaf histological sections were obtained from 76-day-old plants, stained with safranin fast green, and analyzed (Figure 4a). The vascular parenchyma sections and the vascular bundle diameters were larger in H11 plants than in controls, whereas pH11 plants did not show significant differences, except for the pH11-2 line exhibiting shorter vascular bundle diameters (Figure 4). Bulliform cell number and area did not differ among genotypes (Supplementary Figure S6a,b). We obtained a similar result by analyzing the transversal area of the uppermost internode (Figure 4b); H11 plants had larger surfaces than controls and pH11 (Figure 4b). The observation of longitudinal sections of the uppermost internode suggested increased stomatal density in H11 plants (Supplementary Figure S6d). Tiller thickness exhibited a different scenario (Figure 4c); those of controls were wider than those of the H11 and pH11-2 lines, whereas this characteristic did not differ in the pH11-3 line. Although controls showed broader tillers, their vascular bundles were narrower than in H11 plants (Figure 4c), whereas the cell number in the stem thickness did not vary among genotypes (Supplementary Figure S6c).

### 3.5. H11 Flag Leaf Angles Are Insensitive to Brassinosteroids

**Figure 5:**
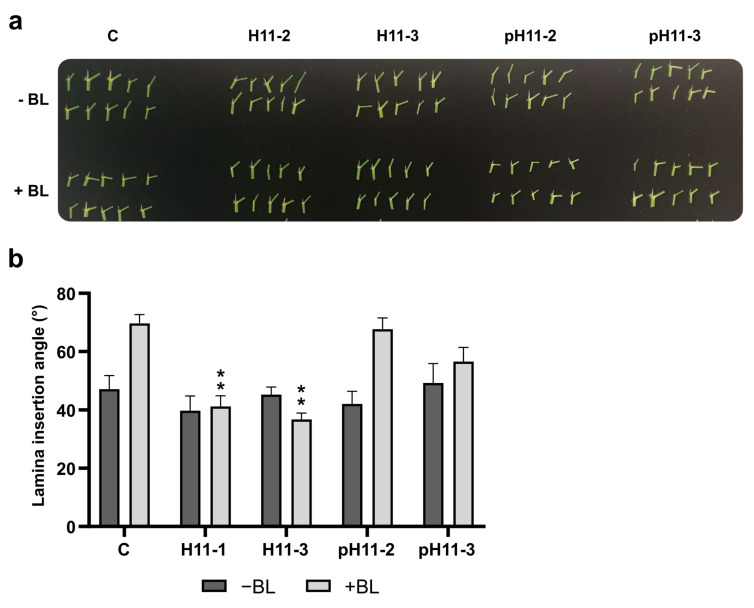
H11 flag leaf angles are insensitive to brassinosteroids. (**a**) Illustrative picture of detached leaf section including lamina joint of control (C), H11 (two independent lines, H11-2 and H11-3), and pH11 (two independent lines, pH11-2 and pH11-3) plants before (upper panel) and after (lower panel) brassinolide treatment; the scale bar represents 1 cm. (**b**) Angle of the lamina joint. The assays were repeated at least twice with N: 10–12/genotype. Error bars represent SEM. Asterisks indicate significant differences (** *p* < 0.01).

Flag leaf architecture is one of the determinants of plant light use efficiency, which is related to a better photosynthetic rate. Shorter angles between the flag leaf and the uppermost internode approach the ideal phenotype, whereas larger ones are undesirable. Angle aperture is positively modulated by brassinosteroids (BRs), whereas ABA has the opposite effect. To evaluate the differential flag leaf angle phenotype in H11 plants and test their sensitivity to BRs, detached plant leaves, including lamina joints, were treated with brassinolide. As expected, wild-type and pH11 plants opened their angles until almost 90°, whereas H11 plants remained with the same aperture, indicating that these plants are insensitive to BRs (Figure 5). We quantified the transcript levels of enzymes participating in BR synthesis and perception in all the genotypes. Surprisingly, they did not show significant differences, except for *OsBRD1*, which was induced in H11 plants (Supplementary Figure S7), suggesting that a post-transcriptional modulation or an alternative molecular mechanism was taking place to trigger this BR insensitivity in H11 plants. Additional genes involved in BR synthesis and signaling were evaluated but were undetectable in our samples.

### 3.6. The Constitutive Expression of the Sunflower HaHB11 Gene Is Necessary to Confer the Differential Traits

**Figure 6:**
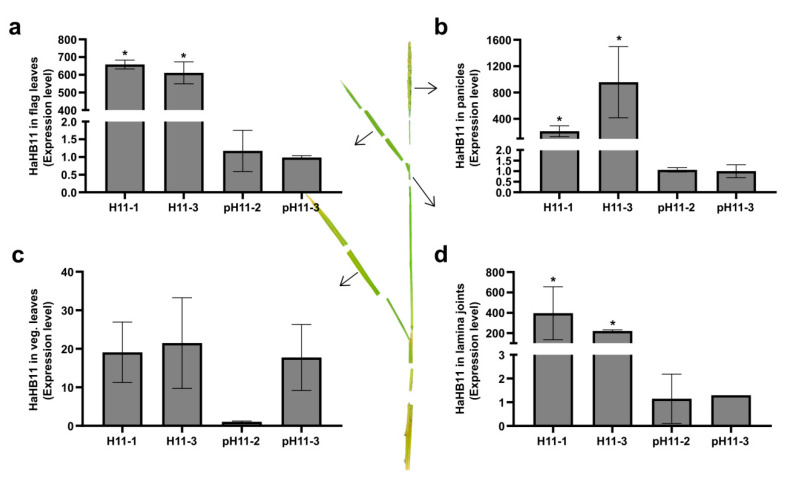
The localized expression of the sunflower *HaHB11* gene is necessary to confer the differential traits. (**a**) *HaHB11* transcript levels evaluated with RT-qPCR in 93-day-old H11 (two independent lines, H11-2 and H11-3) and pH11 (two independent lines, pH11-2 and pH11-3) flag leaves; the same panicles (**b**), vegetative leaves (**c**), and lamina joint (**d**). All the values were normalized to those obtained in pH11-2 plants, which were arbitrarily assigned a value of 1 (one). N: 3/genotype. Error bars represent SEM. Asterisks indicate significant differences (* *p* < 0.05).

The observations described above evidence that *HaHB11* expression driven by the 35S CaMV promoter resulted in the differential traits allowing H11 plants to approach the ideal phenotype, whereas those in which the *HaHB11* promoter caused the expression of the sunflower gene did not significantly differ from controls. Considering that the ideal phenotype comprised several characteristics in different tissues and developmental stages, we wondered in which tissues *HaHB11* expression was necessary. To answer this question, we evaluated *HaHB11* transcript levels in H11 and pH11 plants (Figure 6). While expression levels did not differ in vegetative leaves among these genotypes (except for a lower expression in the pH11-2 line), the differences in flag leaves, panicles, and the lamina joint tissue were enormous. These results indicate, on one hand, that the *HaHB11* promoter does not induce enough expression in rice flag leaves, lamina joint, and panicles to impact the phenotype, and on the other hand, that in those tissues, and not in vegetative leaves, expression is needed and responsible for the differential traits (Figure 6). 

## 4. Discussion

Rice breeding programs use the ideotype approach to improve yield potential. Good results were obtained by combining this approach and intersubspecific heterosis, reaching a grain yield of 12 t ha^−1^ [2]. The ideal phenotype includes moderate tillering capacity; heavy and dropping panicles at maturity; a harvest index of at least 0.55; and specific traits in the top three leaves, such as being erected, large and rolled, and having a sharp flag leaf angle, among other characteristics [31]. Notably, both strategies focus on the same traits. However, yield potential has stagnated at about 10 Mg ha^−1^ in tropical environments since the Green Revolution [31,32]. Here, we show that the expression of a single heterologous gene, the sunflower *HaHB11* gene, achieved excellent results, approaching the ideal phenotype. 

Biomass partitioning was one of the distinctive features of H11 plants, evidenced by the lower tiller/leaf ratio and supported by the evaluation of tiller and leaf biomass/plant, reduced and enhanced in H11 plants compared with their controls, respectively. Moreover, H11 flag leaves were larger and taller (overpassing the panicle height) and exhibited better architecture, evidenced by their erected form. Even though a particular sowing density value was not defined, for japonica varieties, such density varying between 50 and 150 kg/ha did not significantly affect most yield components [25]. Taking into account that Kitaake plants are short and hardly reach 100% light interception, phenotyping at 226 plants/m^2^ (equivalent to 61 kg/ha) would not significantly change when varying this parameter. The reduced tiller capacity, the enhanced uppermost internode area, and the increased vascular bundle diameters both in the uppermost internode and the tillers also helped the differential biomass partitioning in H11 plants. Additionally, the harvest index shown by these plants was around 0.6 (Figure 2i), higher than the looked-for 0.55 [2], whereas Kitaake controls exhibited a value of 0.3 (Figure 2i). Even though the absolute measurements in H11 plants differed from those described for the ideotype [2], all the differential traits pointed in the same direction. The distance with the ideotype can be explained by the background genotype being Kitaake, which is not an elite variety. For example, ideal tiller capacity was established in 270–300 panicles/m2 and increased in our assays, but not in H11 plants, in which it was significantly lower than in controls (Figure 2). Notably, neither the green index nor total flag leaf biomass per plant differed between controls and H11 plants. Although H11 plants exhibited fewer panicles and flag leaves, total biomass was similar, because each flag leaf was larger, heavier, and more erected. These characteristics are likely due to the rolled form and the increased vascular parenchyma section (Figure 4a), in agreement with the traits considered in the ideotype [2]. 

Fewer IAA provision to the uppermost internode and gibberellin deficiency resulted in cell number and elongation decreases, generating a shortened uppermost internode. In this situation, the panicle cannot push and go out of the flag leaf and results in panicle enclosure, blocking normal pollination and reducing seed production [3]. Remarkably, H11 plants exhibited unexpectedly shorter uppermost internodes than their controls; however, they did not show panicle enclosure, indicating that this trait was somehow compensated for. EUI1 is a GA-deactivating enzyme; when the encoding gene was overexpressed, it triggered a GA-deficient dwarf phenotype [33,34]. The HD-Zip TF HOX12 represses *EUI1*, enhancing panicle exertion [17]. It is tempting to speculate that HaHB11 can dimerize with HOX12, inhibiting its natural function.

Plant architecture defines its ability to intercept light. In this sense, the erected leaves allow light to reach the whole canopy, leading to better net photosynthesis. H11 plants presented a differential architecture compared with controls and pH11 ones, distinguishable due to their sharper tiller and flag leaf angles, and increased leaf area supported by the double length of these leaves. Moreover, the rolled and erected leaves contributed to a compact phenotype. Particularly, flag leaf width [35] and length [2] were positively correlated with yield per plant, probably helping H11 plants to enhance seed yield.

Abaxial leaf rolling is an important agronomic trait. Interestingly, the suppression of Roc5 (Rice outermost cell-specific gene 5), an ortholog of the Arabidopsis GLABRA2 gene (a class IV HD-Zip), resulted in abaxial leaf rolling, whereas its overexpression led to adaxially rolled leaves. These characteristics went together with bulliform cell number and size increases [36], impacting seed yield. H11 plants exhibited rolled leaves, and interestingly, Roc5 was induced in their panicles, albeit not in the flag leaves, suggesting that the rolling can be somehow the result of this HD-Zip IV gene modulation.

Erected leaves make more efficient light capture and penetration to the whole canopy possible, improving photosynthesis and nitrogen storage for grain filling, allowing higher planting density to be obtained, which triggers increased grain yield [32,37]. Although H11 plants exhibited this characteristic plus sharper insertion angles, they did not differ in light interception. This fact was intriguing; however, the enhanced yield can be explained by improved partitioning, favored by better plant architecture. The differences in their vascular system morphology, tiller and leaf vascular bundles with larger diameters, and shorter uppermost internodes could allow photosynthates to be better transported to panicles. Moreover, the number of panicles per tiller was around 1 in H11 plants and 1.5 in controls (Supplementary Figure S3e). Notably, total biomass/plant at harvest did not significantly differ among genotypes, and H11 plants had fewer tillers, suggesting that such plants concentrated their resources, performing more efficient although energy-consuming photosynthate transport, which negatively impacted dry net biomass accumulation, as evidenced by the significantly increased grain yield (Figure 2).

The rice HD-Zip I genes Oshox12 and Oshox14 are the closest homologs of Vrs1, and a recessive mutation in Vrs1 (HvHox1) changes two-rowed barley to six-rowed barley, which considerably improves yield [38,39]. *OsHOX12* and *OsHOX14* were highly expressed in developing panicles, and their overexpression generated reduced panicle length and a dwarf phenotype. Moreover, the lines overexpressing *OsHOX14* showed a deficiency in panicle exertion [40]. We showed that the ectopic and constitutive expression of *HaHB11* generated a partially contrasting phenotype. Even though H11 plants exhibited shorter uppermost internodes below this internode, the plant was higher, compensating for the total height, similar to controls. Unfortunately, *Oshox12* and *Oshox14* mutants or edited plants have not been described so far, but it is tempting to speculate that the sunflower gene could be heterodimerizing with these TFs, avoiding deficiency in panicle exertion. Another possibility is the interaction with OsHOX6, the closest rice HaHB11 TF; however, no expression or functional information about this rice gene supporting this hypothesis is available. Heterodimerization among HD-Zip I TFs has already been reported, even with members from different species [11,41]. Alternatively, HaHB11 can bind the target sequences of rice TFs, blocking the possibility of OsHOX6, -12, and -14 acting in this developmental stage. Further profound studies will be necessary to corroborate these hypotheses. 

Lamina joint inclination is antagonistically modulated by brassinosteroids and high ABA concentrations, whereas low ABA concentrations have an effect opposite to that exerted by higher ones [42], stimulating and repressing angle aperture, respectively [43]. The brassinosteroid-deficient mutant *osdwarf4* exhibited sharper angles and erected leaves [44]. Notably, H11 plants were insensitive to brassinosteroids in agreement with their erected leaves and sharper lamina joint angle phenotype. However, among the evaluated genes associated with these characteristics, only *OsBRD1*, involved in brassinosteroid biosynthesis, was induced in H11 plants (Supplementary Figure S7). We were not able to detect *BRD-2* or *D11*, also involved in the synthesis, or *OsBZR-1*, *OsGSK2*, *OsLIC*, or *OsDLT,* participating in BR signaling, probably due to sample dilution, because we took one linear centimeter from the flag leaf and the expression of these genes only localized in a few cells of the lamina joint. On the other hand, we expected the repression of *OsBRD1*, but it showed induction. It is possible that H11 plants sensed the lack of BRs, and in consequence, activated this gene in vain to compensate for the situation (Supplementary Figure S7).

All the differential characteristics shown by H11 plants that allowed them to approach the ideotype required the expression of the sunflower gene in the reproductive stage, particularly in rice flag leaves, lamina joint tissue, and panicles, where the *HaHB11* promoter does not drive expression in these plants.

It is important to note that H11 plants had more green areas when they switched to anthesis because flowering was delayed. Notably, the total life cycle was similar to that of controls because their panicles accumulated biomass faster. Moreover, these plants exhibited enhanced photosynthate transport to leaves. In agreement with this, longer flowering times have been proposed, as additional physiological traits may result in further improvements [45]. 

**Figure 7:**
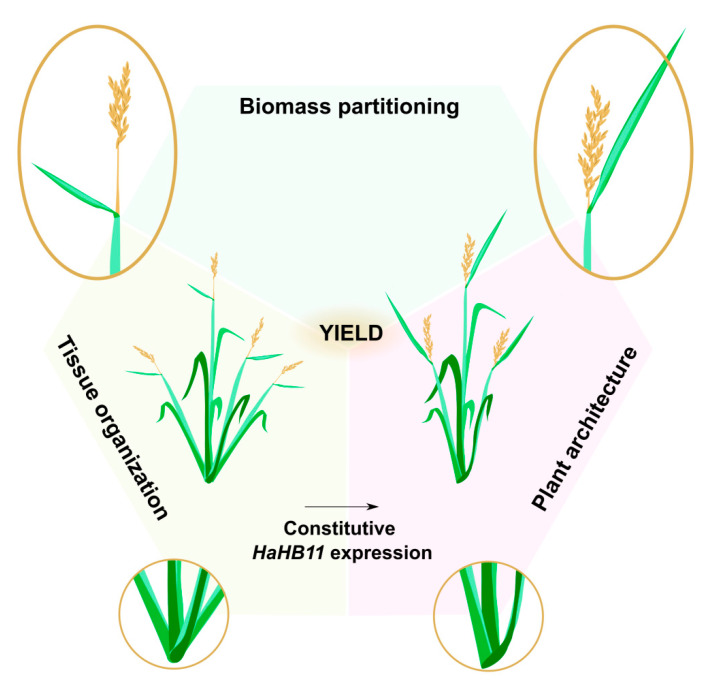
Proposed model of *HaHB11* action allowing rice plants to approach their ideal phenotype.

*HaHB11* has become a biotechnological tool to improve rice yield, either with its overexpression or by elucidating the molecular mechanism by which this sole gene can confer all the differential phenotypes approaching the rice ideotype. Future studies will be necessary to investigate the latter alternative (Figure 7). 

Summary of the main differential traits exhibited by H11 plants compared with controls. Enlargements of some traits are shown in circles.

## Data Availability

All the data corresponding to this study was included in the article or in the Supplementary Material.

## Supplementary Materials

The following are available online at https://www.mdpi.com/article/10.3390/biom13050826/s1, Figure S1: OsHOX6, the closest rice HD-Zip I member, significantly differs from HaHB11, Figure S2: Biomass partitioning differs between H11 plants and their controls, Figure S3: H11 plants exhibit larger panicles exhibiting more branches than controls, Figure S4: H11 plants exhibit longer tillers, shorter uppermost internodes, and sharper flag leaf angles than their controls, Figure S5: *OsROC5* is upregulated in H11 panicles, Figure S6: The surface of bulliform cells is enhanced in H11 plants, Figure S7: OsBRD-1, encoding a key enzyme involved in brassinosteroid synthesis, is induced in HB11 plants, Table S1: Dates of sowing, waterlogging, anthesis, and harvesting in the five field assays, Table S2: Oligonucleotides used for cloning or RT-qPCR, Table S3: Trait values measured in each campaign, Table S4: Statistical analyses of the trials.
