# Supplementary material for: The Transcription Factor HaHB11 Boosts Grain Set and Yield in Rice Plants, Allowing Them to Approach Their Ideal Phenotype
Source: Biomolecules. 2023 May 12;13(5):826. doi: 10.3390/biom13050826 (PMC10216415; doi:10.3390/biom13050826)
Supplement: Supplementary file 1 [file biomolecules-13-00826-s001.zip › biomolecules-2301706-final-supplementary.pdf]

Supplementary Materials: The following are available online at https://www.mdpi.com/article/10.3390/biom13050826/s1,

a

Score	Expect	Method	Identities	Positives	Gaps
131 bits(330)	4e-43	Compositional matrix adjust.	61/94(65%)	79/94(84%)	0/94(0%)

```
Query  20   RRFSDEQIKSLESVFKRENKLEPRKKVEMARELGLHPRQVAIWFQNRRARWKSKQVEQDY  79
            +RFS+EQIKSLES+F  + KLEPR+K+++ARELGL PRQVAIWFQN+RARWKSKQ+E++Y
Sbjct  32   KRFSEEQIKSLESMFATQTKLEPRQKLQLARELGLQPRQVAIWFQNKRARWKSKQLEREY  91

Query  80   YNLKADYDTLAHRFESLKKEKHALLHQVSQLKEL  113
              L+ DYD L   +ESLKKEK AL+ Q+ +L E+
Sbjct  92   SALRDDYDALLCSYESLKKEKLALIKQLEKLAEM  125
```

b

```
HaHB11          1 -------------------MAENSSSSIERKKSKKHNNRRFSDEQIKSLE     31
                                     .......:.:|||       |||:|||||||
OsHOX6          1 MDGEEDSEWMMMDVGGKGGKGGGGGGAADRKK-------RFSEEQIKSLE     43

HaHB11         32 SVFKRENKLEPRKKVEMARELGLHPRQVAIWFQNRRARWKSKQVEQDYYN     81
                  |:|..:.|||||:|:::||||||.||||||||||:||||||||:|::|..
OsHOX6         44 SMFATQTKLEPRQKLQLARELGLQPRQVAIWFQNKRARWKSKQLEREYSA     93

HaHB11         82 LKADYDTLAHRFESLKKEKHALLHQVSQLKEL-----DSGSENGGELKNG    126
                  |:.|||.|...:|||||||.||:.|:.:|.|:     ....:|.|:
OsHOX6         94 LRDDYDALLCSYESLKKEKLALIKQLEKLAEMLQEPRGKYGDNAGD----    139

HaHB11        127 NSSSGPLEYMQGDKLV----------------------------------    142
                  ::.||.:..|:.::.|
OsHOX6        140 DARSGGVAGMKKEEFVGAGGAATLYSSAEGGGTTTTTTAKLMPHFGSDDV    189

HaHB11        143 -----------------------SEEEEEERHENLDMASLF----DQSCS    165
                                         :..|....|::.:..|.:    :|:||
OsHOX6        190 DAGLFLRPSSQHHPPPPHAGAGFTSSEPAADHQSFNFHSSWPSSTEQTCS    239

HaHB11        166 N--WWDIWSSNS*    176
                  :  ||:..|.
OsHOX6        240 STPWWEFESE---    249
```

Figure S1. OsHOX6, the closest rice HD-Zip I member, significantly differs from HaHB11

a) Blast result and (b) global alignment of OsHOX6 and HaHB11.

Identity: 79/263 (30.0%)

Similarity: 115/263 (43.7%)

Gaps: 101/263 (38.4%)

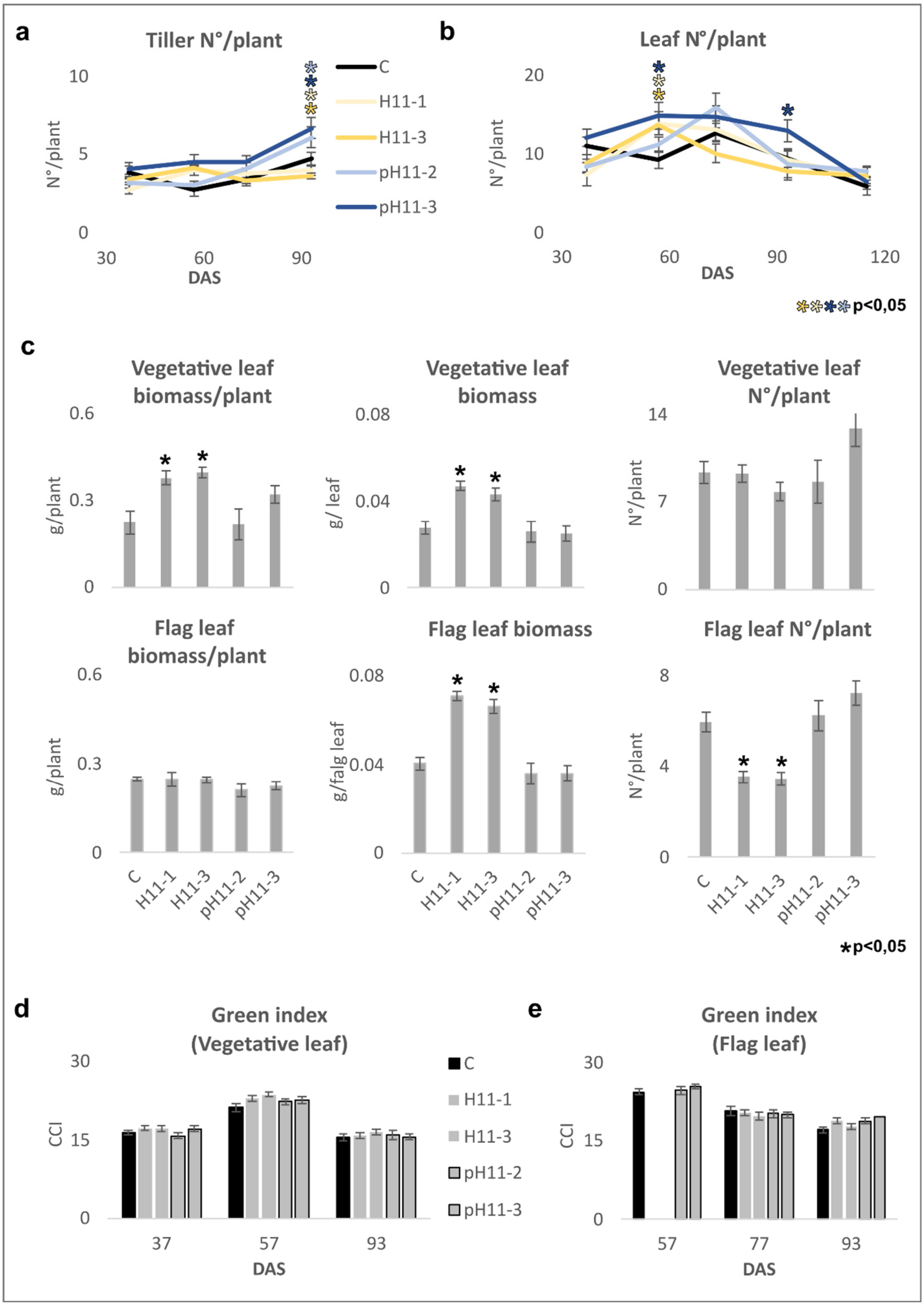

Figure S2. Biomass partitioning differs between H11 plants and their controls

(**a**) Number of tillers per plant; (**b**) Number of leaves per plant; (**c**) Vegetative leaf biomass per plant, vegetative leaf biomass per flag leaf, number of vegetative leaves per plant, flag leaf biomass per plant, individual flag leaf biomass, flag leaf number per plant.

These analyses were carried out with 93-day-old plants; (**d**) Green index in the vegetative leaf; (**e**) in the flag leaf. Assays were repeated in two campaigns with N: 18-24/genotype. Wild-type Kitaake plants were used as controls (C) and graphed in black color, whereas H11 and pH11 plants were represented in grey and gray with black borders, respectively. Error bars represent SEM. Asterisks indicate significant differences doing ANOVA test followed by Tukey post-hoc test (* $P < 0.05$).

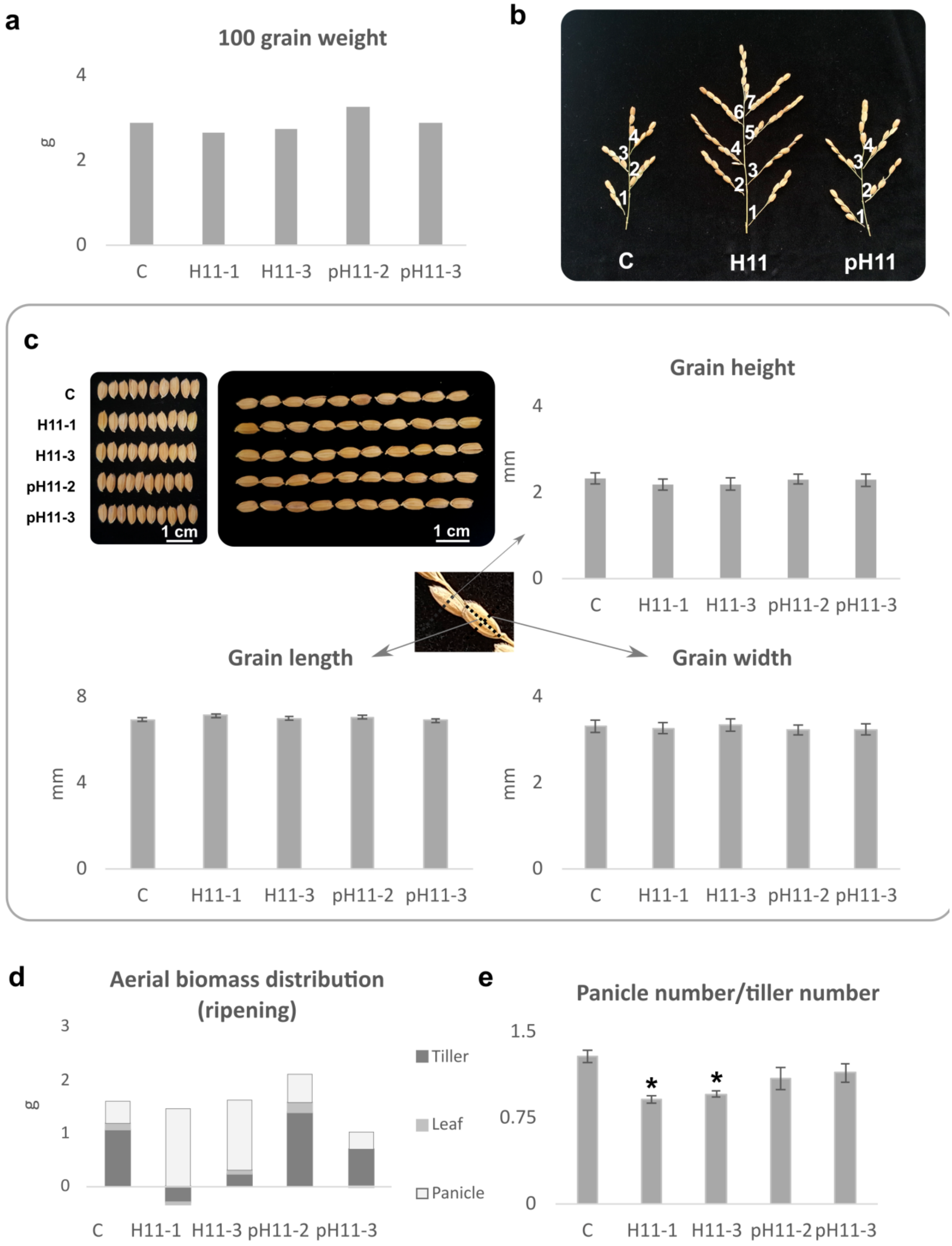

Figure S3. H11 biomass at ripening significantly exceeds that of controls and pH11 plants, whereas grain associated traits did not differ between genotypes

(**a**) 100 grains weight; (**b**) Illustrative picture of panicles of control, H11, and pH11 plants. The white numbers indicate the position in the panicle; (**c**) Illustrative pictures of grains of control, H11 (two independent lines H11-2 and H11-3), and pH11 (two independent

lines pH11-2 and pH11-3) plants. White bars represent 1 cm, grain height (upper right panel), grain length (lower left panel), and grain width (lower right panel); (**d**) Aerial biomass distribution 16 days after flowering. Values represent aerial biomass of tillers, leaves, and panicles 16 days after flowering minus the biomass of the same organs taken at flowering and expressed in grams; (**e**) Number of panicles per tiller. Error bars represent SEM. An ANOVA test was performed, followed by a Fisher LSD post-hoc test. * $P < 0.05$

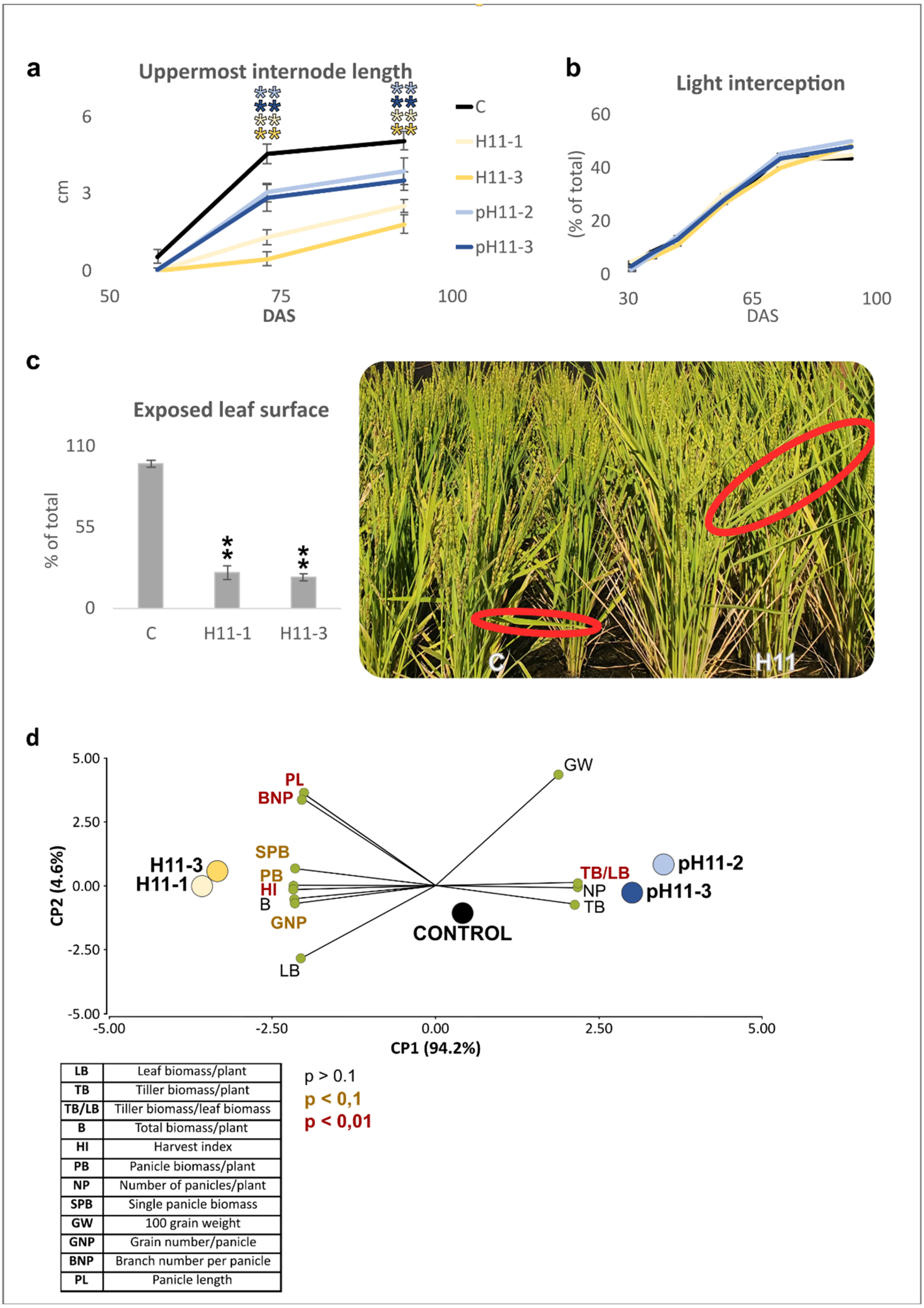

Figure S4. H11 plants exhibit longer tillers, shorter uppermost internodes, and sharper flag leaf angles than their controls

(**a**) Uppermost internode length; (**b**) Light interception; (**c**) Exposed leaf surface and illustrative pictures of the plants, marking in red circles flag leaves; In **(a), (b),** and **(c),**

error bars represent SEM. Asterisks indicate significant differences doing a Student's t-test (*$P< 0.05$; **$P< 0.01$). (**d**) Principal component analysis considering the parameters (signaled in light green) evaluated during the five trials for control (black circle), H11 (yellow and light yellow circles for each event, respectively), and pH11 (blue and light blue circles for each event, respectively) plants. The table at the bottom indicates the abbreviations used in the PCA analysis. P-values representing significant differences are signaled at the right of the table in color.

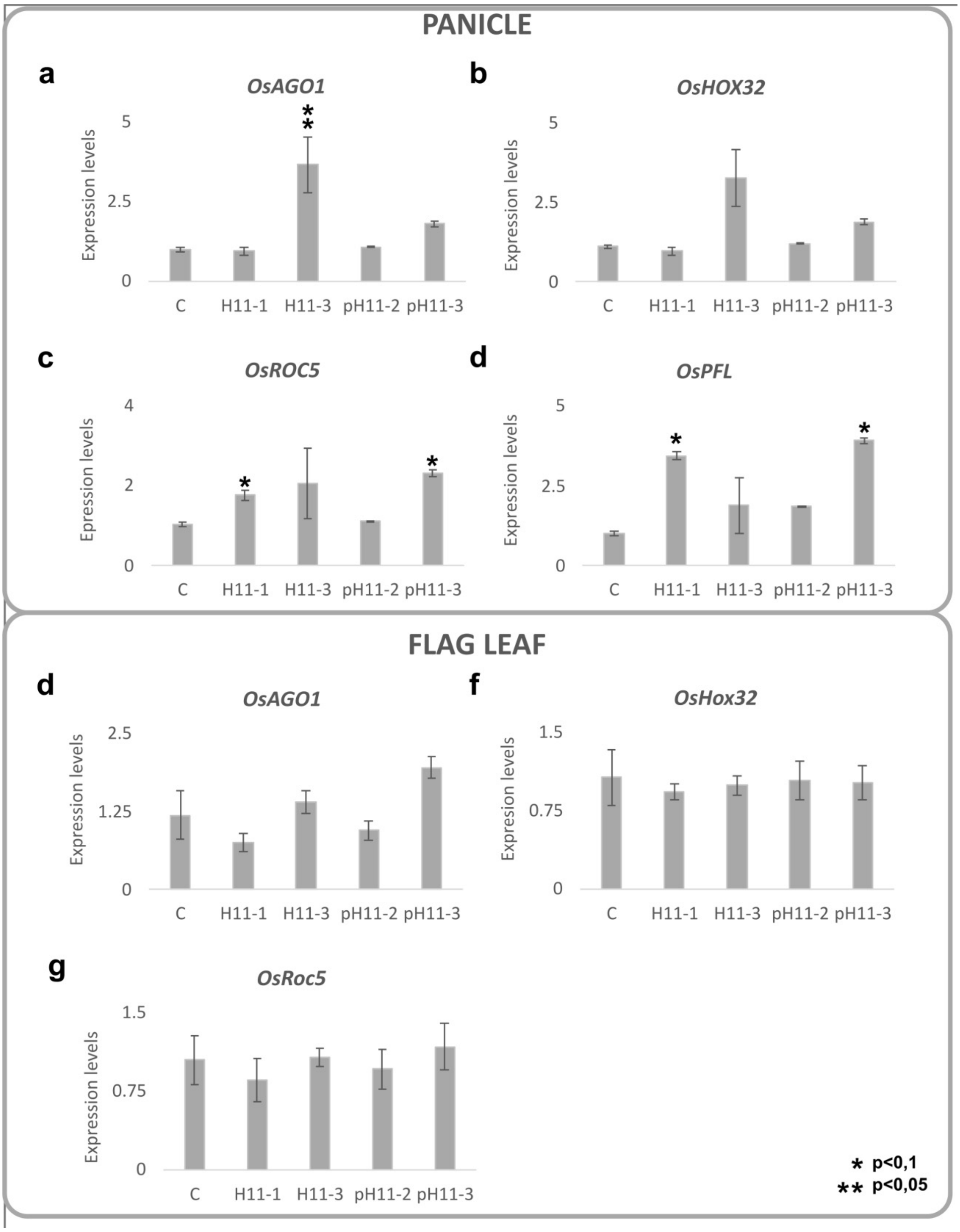

Figure S5. ***OsROC5*** **is upregulated in H11 panicles**

Transcript levels of *OsAGO1* (**a**), *OsHOX32* (**b**), *OsROC5* (**c**), and *OsPFL* (**d**) in panicles of the five genotypes (Control (C), H11-2, H11-3, pH11-2, and pH11-3). Transcript levels of *OsAGO1* (**e**), *OsHOX32* (**f**), *OsROC5* (**g**) in flag leaves of the same plants. The values were normalized with those measured in controls, arbitrarily assigned a value of 1 (one). Error bars represent SEM. Asterisks indicate significant differences doing a Student's t-test (** P < 0.05, * P < 0.1).

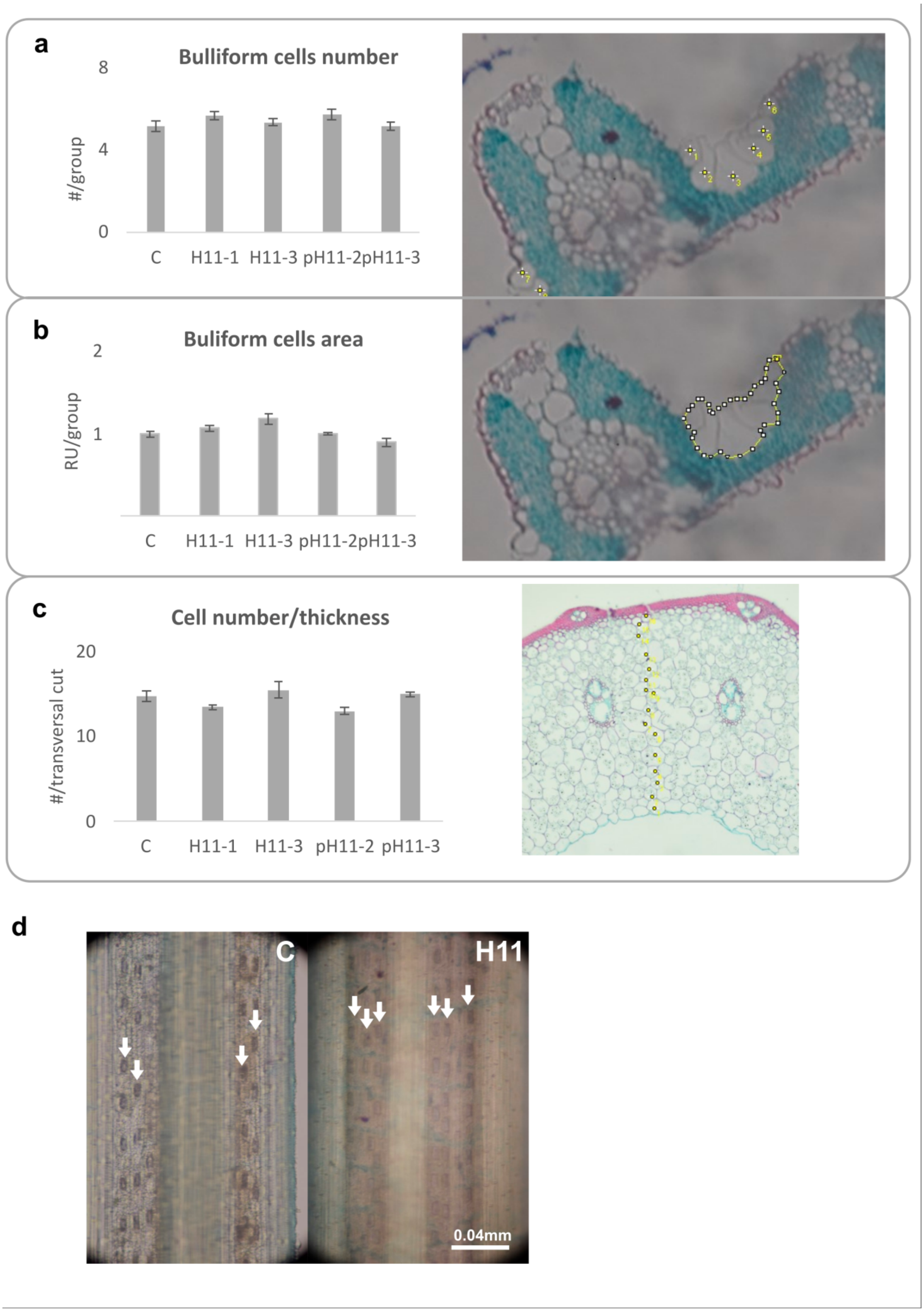

Figure S6. The surface of bulliform cells is enhanced in H11 plants

Left panels: bulliform cell number (**a**); bulliform cell area (**b**); cell number/tiller thickness ratio (**c**). Right panels: illustrative pictures showing the quantified parameter. (**d**)

Transversal peeling of uppermost internode sections. White arrows signal stomata. Error bars represent SEM.

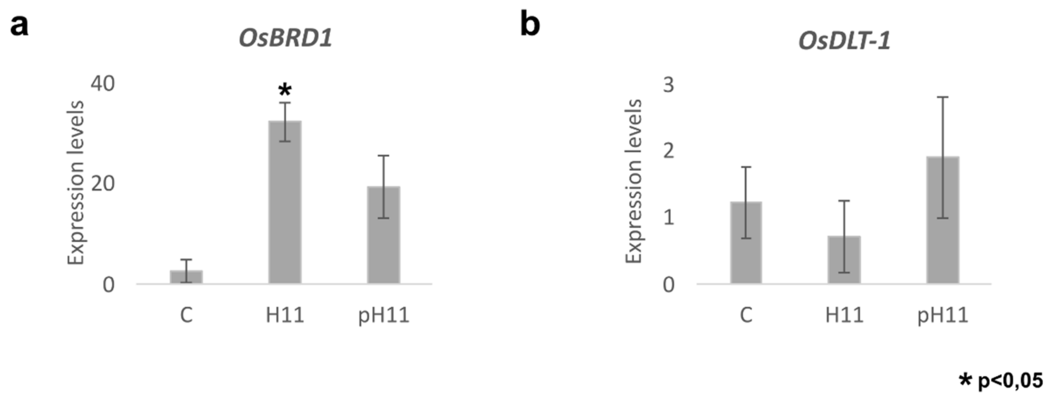

Figure S7. ***OsBRD-1*****, encoding a key enzyme involved in brassinosteroid synthesis, is induced in HB11 plants**

Transcript levels of *OsBRD-1* (**a**) and *OsDlT-1* (**b**) in control, H11 and pH11 plants. Samples were taken of 1 cm linear segment detached leaf including lamina joint. The values were normalized with those measured in controls, arbitrarily assigned a value of 1 (one). Error bars represent SEM. Asterisks indicate significant differences doing an ANOVA test, followed by a Fisher LSD post-hoc test. *P< 0.05

In the attached excel file, there are four tables including these information

Table S1. Dates of sowing, waterlogging, anthesis, and harvesting of the five field assays

Table S2. Oligonucleotides used for RT-qPCR and genes IDs.

Table S3. Trait values measured in each campaign

The Table shows the sowing date and the data corresponding to all the evaluated traits, including light interception for five and two campaigns performed with H11 and pH11 genotypes, respectively. The means and SEM for each parameter are indicated on the right of each column.

Table S4. Statistical analyses of the trials

The data shown correspond to the analysis of the campaigns carried out with H11 and pH11 genotypes during the seasons 2017-2018, 2018-2019, 2019-2020, 2020-2021, and 2021-2022. Three biological replicates per genotype (H11, pH11, IRGA417, and control), and campaign were evaluated. An ANOVA analysis was performed considering the campaign and each event for H11 or pH11 lines. Then, taking the genotype as the classification variable and each of the traits as the dependent variable, data were compared using Tukey (p-values ("p<0,1 *; p<0,05 **, and p<0,01 ***"). IRGA 417 is a commercial genotype used as a control in two out of five assays.
